# The nose is the best niche for detection of experimental pneumococcal colonisation in adults of all ages, using nasal wash

**DOI:** 10.1038/s41598-021-97807-1

**Published:** 2021-09-14

**Authors:** Elissavet Nikolaou, Esther L. German, Annie Blizard, Ashleigh Howard, Lisa Hitchins, Tao Chen, Jim Chadwick, Sherin Pojar, Elena Mitsi, Carla Solórzano, Syba Sunny, Felicity Dunne, Jenna F. Gritzfeld, Hugh Adler, Jason Hinds, Katherine A. Gould, Jamie Rylance, Andrea M. Collins, Stephen B. Gordon, Daniela M. Ferreira

**Affiliations:** 1 Department of Clinical Sciences, Liverpool School of Tropical Medicine, 1st Daulby Street, Liverpool, L7 8XZ UK; 2 Medical Microbiology, Royal Liverpool University Hospital, Liverpool, UK; 3 Infection and Immunity Research Institute, St George’s University London, London, UK; 4 College of Medicine, Queen Elizabeth Central Hospital, Blantyre, Malawi; 5 Present Address: Institute of Inflammation and Ageing, University of Birmingham, Birmingham, UK; 6 Present Address: Institute of Life Course and Medical Sciences, University of Liverpool, Alder Hey Children’s NHS Foundation Trust Hospital, Liverpool, UK

**Keywords:** Applied microbiology, Bacteria, Bacteriology, Clinical microbiology, Infectious-disease diagnostics, Pathogens, Microbial ecology

## Abstract

Previous studies have suggested that the pneumococcal niche changes from the nasopharynx to the oral cavity with age. We use an Experimental Human Pneumococcal Challenge model to investigate pneumococcal colonisation in different anatomical niches with age. Healthy adults (n = 112) were intranasally inoculated with *Streptococcus pneumoniae* serotype 6B (Spn6B) and were categorised as young 18–55 years (n = 57) or older > 55 years (n = 55). Colonisation status (frequency and density) was determined by multiplex qPCR targeting the *lytA* and *cpsA*-6A/B genes in both raw and culture-enriched nasal wash and oropharyngeal swab samples collected at 2-, 7- and 14-days post-exposure. For older adults, raw and culture-enriched saliva samples were also assessed. 64% of NW samples and 54% of OPS samples were positive for Spn6B in young adults, compared to 35% of NW samples, 24% of OPS samples and 6% of saliva samples in older adults. Many colonisation events were only detected in culture-enriched samples. Experimental colonisation was detected in 72% of young adults by NW and 63% by OPS. In older adults, this was 51% by NW, 36% by OPS and 9% by saliva. The nose, as assessed by nasal wash, is the best niche for detection of experimental pneumococcal colonisation in both young and older adults.

## Introduction

The natural flora of the human upper respiratory tract (URT) is in constant interaction with the external environment, resulting in a diverse ecology of microorganisms colonising epithelial surfaces in the oral and nasal cavities and oropharynx^1^. *Streptococcus pneumoniae* (Spn, pneumococcus) can proliferate and establish colonisation as a non-pathogenic symbiont, which is rarely symptomatic for the host, particularly in adults^2–4^. Pneumococcus can however migrate to other organs, resulting in pneumonia, meningitis, and bacteraemia, causing significant morbidity and mortality worldwide, especially in the very young and very old.

Colonisation of the URT has been shown to be the source of, and pre-requisite for, pneumococcal disease^5^ and its transmission throughout the community^6^. Accurate detection of pneumococcal colonisation is crucial to assessing disease potential, as well as direct and indirect impact of vaccines. The World Health Organisation (WHO) recommends the use of nasopharyngeal swabs for pneumococcal colonisation detection in children and both nasopharyngeal and oropharyngeal swabs (OPS) in adults^7,8^. WHO recommendations were based on culture-based methods and more recent studies using sensitive molecular methods have encouraged other sampling methods such as saliva or nasal wash^9,10^, due to improved comfort and pneumococcal detection.

Many studies have investigated pneumococcal colonisation prevalence in the first few years of life, showing that the nasopharynx of 40–95% of young children^11,12^ is naturally colonised with pneumococcus. With the onset of adulthood, falling pneumococcal disease rates are accompanied by decreased nasopharyngeal colonisation rates at 10–25%^13–16^. In older adults, a population at increased risk of pneumococcal disease, colonisation prevalence and density are reported to be lower, but study results are heterogeneous with some reporting very low colonisation rates of 1.9–4.2%^17–19^. Our recent systematic review analysed 29 studies, 18 with participant-level data (representing 6290 participants) and reported prevalence of detected pneumococcal colonisation of 0–39% by conventional culture methods and 3–23% by molecular methods^20^.

Various theories have been put forward to explain the paradox of increased disease risk with decreased colonisation prevalence, including a more transient colonisation dynamic in older adults^18^, the poor sensitivity of conventional culture methods to detect low density colonisation in polymicrobial samples^21,22^ and a transition of the pneumococcal niche from the nasopharynx to the oral cavity with ageing^21^.

The Experimental Human Pneumococcal Colonisation (EHPC) model is a safe, reproducible, and controlled method of studying colonisation dynamics in adult human participants, as the challenge dose and time of exposure is known^23^. We have recently expanded this model to older adults and reported that experimental colonisation was established in 39% of participants (25/64) with no adverse events. Colonisation occurred in 47% (9/19) of participants aged 50–59 and in 42% (13/31) of those aged 60–69 compared to 21% (3/14) in those aged ≥ 70 years. Colonisation density was similar between old and younger adults^24^.

We used 780 nasal, oropharyngeal and saliva samples to define precisely whether the niche of experimental pneumococcal colonisation changes in adults with increasing age. Association with bacterial density was also analysed.

## Materials and methods

### Clinical trial design, participant cohorts and sample analysis

The methodology and inclusion/exclusion criteria for EHPC studies have been previously described^25^. Briefly, participants were healthy adults aged ≥ 18 years with no major risk factors for pneumococcal disease, colonisation, or transmission (such as: cigarette smoking; close contact with children aged < 5 years; healthcare work or caring responsibilities; steroid therapy and respiratory or immunosuppressive comorbidities). Studies were conducted in accordance with the Declaration of Helsinki and Good Clinical Practice procedures. All participants provided written informed consent and underwent a safety screening. Studies were submitted to and approved by a local NHS Research Ethics Committee. In this study, samples have been sourced from two separate EHPC clinical studies. The young vaccine study was a double-blind, randomised controlled trial conducted between September 2013–April 2014 investigating the impact of PCV-13 vaccination on experimental pneumococcal colonisation in adults aged 18–50 (NHS REC number 12/NW/0873, ISRCTN number 45340436 registered 18/11/2013)^26^. In the present analysis, we include participants from the control arm only (vaccinated with Hepatitis A vaccine rather than PCV-13). The older adults study was an observational study of the effect of age on experimental pneumococcal colonisation in adults aged 50–80 conducted between June 2016–February 2018 (NHS REC number 16/NW/0031, ISRCTN number 10948363 registered 08/11/2016)^24^. In total, 112 participants were analysed. The sample sets can be combined because the inoculation dose and strain, as well as the methods for inoculation, nasal wash collection and processing remained identical. Furthermore, pneumococcal colonisation rates and serotype distribution in healthy adults in Liverpool appears to be relatively stable, based on data collected between 2010–2017^27^. The low prevalence of naturally occurring Spn6 provides reassurance that detected Spn6B is the inoculated strain. Using the full sample set, participants were split into young 18–55 years (n = 57) and older adults > 55 years (n = 55), in keeping with the cut-off used by Marrie et al.^28^.

In both studies, participants were inoculated with 80,000 colony-forming units (CFU) per nostril of live Spn6B pneumococcus (BHN418, GenBank accession number ASHP00000000.1)^26^. Nasal wash (NW) samples were collected before inoculation to screen for pneumococcal colonisation acquired from community. Depending on the study, NW, oropharyngeal swabs (OPS) and saliva samples were then collected post pneumococcal exposure. In the young vaccine study, NW and OPS samples were collected on days 2, 7, 14 (only culture-positives) and 21 post exposure. In the older adults study, NW, OPS and saliva samples were collected on days 2, 7, 9, 14, 22 (only culture-positives) and 29 post exposure. Pneumococcal colonisation status in both studies was determined by NW culture (not discussed here) and by multiplex real-time polymerase chain reaction (qPCR) targeting the *lytA* and *cpsA*-6A/B genes in raw and culture-enriched NW, OPS, and saliva samples. The raw and culture-enriched NW, OPS, and saliva qPCR data from days 2, 7 and 14 (covered in both studies) were included in this study.

NW collection was performed as previously described^25^. Briefly, 20 mL of 0.9% sodium chloride solution in total (10 mL saline per nostril) was introduced using a syringe and held for a few seconds in the participant’s nose before being expelled into a sterile container. NW was centrifuged at 4000 rpm for 10 min. Supernatant was collected and pellet was resuspended in STGG (Skimmed milk-Tryptone-Glucose-Glycerine) 20% glycerol before storage at − 80 °C. OPS samples were collected in 1 mL STGG and stored at − 80 °C, until further use. Saliva samples were collected using the salivette device (Sarstedt, UK). Following collection, each salivette was centrifuged at 4000 rpm for 3 min at 4 °C and after measuring the volume of saliva liquid retrieved, both pellet and supernatant were re-suspended in equal volume of STGG 50% glycerol and stored at -80 °C, until further use.

### Preparation of raw and culture-enriched NW, OPS, and saliva samples

Before DNA extraction, samples were thawed for 30 min at room temperature and vigorously vortexed for 20 s. 300 µL raw NW pellet, 200 µL raw OPS and 300µL raw saliva aliquots were prepared. For culture-enrichment, 50 µL of NW pellet, OPS or saliva samples were plated on Columbia blood agar supplemented with 5% horse blood and 80μL gentamicin 1 mg/mL. Plates were incubated overnight at 37 °C in 5%CO_2_. Following incubation, 2 mL of STGG 20% glycerol was added onto each plate and microbial growth scraped off. 300 µL culture-enriched NW, 200µL culture-enriched OPS and 300 µL culture-enriched saliva aliquots were prepared. Both raw and culture-enriched NW, OPS and saliva aliquots were stored at − 20 °C until DNA extraction took place.

### DNA extraction on raw and culture-enriched NW, OPS, and saliva samples

Bacterial genomic DNA was extracted from raw and culture-enriched NW, OPS, and saliva samples. On the day of the extraction, prepared aliquots were thawed at room temperature and vigorously vortexed for 20 s. Samples were pelleted at 20,238×*g* for 10 min. Following centrifugation, 300μL of lysis buffer with protease (Agowa Mag mini-DNA extraction kit; LGC Genomics, Germany), 100 μL of zirconium beads (diameter of 0.1 mm), and 300μL of phenol pH 8.0 (toxic, performed in a cabinet with charcoal filter) were added to the pellets. Samples were mechanically disrupted at 50 Hz for 3 min in a tissue homogenizer followed by 3 min on ice, twice. The samples were then centrifuged for 10 min at 9,391×*g*, and the upper aqueous phase was transferred to a sterile 1.5 mL Eppendorf tube pre-filled with 600μL binding buffer and 10μL magnetic beads. The samples were incubated in a mixing machine (~ 265 rpm) for 1 h at room temperature, then washed twice with 200μL of wash buffers 1 and 2. Magnetic beads were dried at 55 °C for 10 min, eluted in 63μL of elution buffer and stored at -20 °C until further use.

### Quantification of pneumococcal DNA by multiplex qPCR in NW, OPS, and saliva pellet samples

We used a multiplex qPCR targeting the *lytA*^29^ and *cpsA*-6A/B^30^ genes as previously described^31^. The reaction mixture of 25µL contained 0.6 µM of each *lytA* primer, 0.3 µM of *lytA* probe, 0.4 µM of each *cpsA*-6A/B primer, 0.2 µM of *cpsA*-6A/B probe, 12.5 µM of Taqman Gene Expression Master Mix (Applied Biosystems, USA) and 2.5µL of extracted DNA. The qPCR reaction was run on a Mx3005P machine (Agilent Technologies, USA) on the following programme: 10 min at 95 °C followed by 40 cycles of 15 s at 95 °C and 1 min at 60 °C. For standard curve, Spn6B DNA was extracted using the QIAamp DNA mini kit (Qiagen, Germany) and serially diluted 1:10 from 4.14 × 10^6^ copies in 2.5 µL. A sample was considered positive if duplicates had a CT value less than 40.

### Quantification and Statistical analysis

Statistical analysis was performed by GraphPad Prism version 5.0 for Windows, GraphPad Software, USA, www.graphpad.com. Contingency tables were used to assess the differences in the pneumococcal frequency in raw and culture-enriched extracted samples in both niches in each age group and in the colonisation frequency between the two niches in each age group or between the two age groups on all study days. The association was tested using Fisher’s exact test and considered significant if *P* < 0.05 (two-sided). Unpaired t-tests were used to compare colonisation densities in experimentally colonised participants, calculated from *cpsA-6A/B* gene copies in raw samples, between niches and age groups. Samples that were negative in the raw sample but positive in the corresponding culture-enriched sample were imputed a density of 1 copy/ml. A Generalized Linear Model (GLM) with binomial distribution was also used to explore the relationship between age and pneumococcal frequency in both niches.

## Results

### Culture-enrichment increased pneumococcal detection in all sample types in both age groups

To detect pneumococcal presence, DNA was extracted from both raw and culture-enriched samples. Samples positive for both *lytA* and *cpsA-6A/B* genes were defined as Spn6B + , whereas those positive only for the *lytA* gene as *lytA* + .

For young adults (n = 57, Supplementary Table S1), 147 NW samples (57 D2, 57 D7 and 33 D14) and 146 OPS samples (57 D2, 57 D7, 32 D14) were assessed. 94/147 (64%) NW samples were Spn6B + :13 only when analysed raw, 20 only when culture-enriched, and 61 by both methods (Supplementary Table S2A: *P* = 0.25 Fisher’s test). 79/146 (54%) OPS samples were Spn6B + : 45 only when culture-enriched and 34 by both methods (Supplementary Table S2B: *P* < 0.0001 Fisher’s test). Culture-enrichment increased the detection of SPN6B + samples in both niches with significant difference in OPS samples.

For older adults (n = 55, Supplementary Table S1), 163 NW samples (55 D2, 55 D7 and 53 D14), 163 OPS samples (55 D2, 55 D7 and 53 D14) and 161 saliva samples (55 D2, 54 D7, 52 D14) were assessed. 57/163 (35%) NW samples were Spn6B + : 3 only when analysed raw, 20 only when culture-enriched, and 34 by both methods (Supplementary Table S3A; *P* = 0.0001 Fisher’s test). 39/163 (24%) OPS samples were Spn6B + : 1 only when analysed raw, 25 only when culture-enriched, and 13 by both methods (Supplementary Table S3B: *P* < 0.0001 Fisher’s test). 9/161 (6%) saliva samples were Spn6B + : 5 only when culture-enriched and 4 by both methods (Supplementary Table S3C: *P* = 0.0294 Fisher’s test). Culture-enrichment significantly increased the detection of Spn6B + samples in all three niches.

There was greater additional benefit in culture-enriching oropharyngeal samples (OPS and saliva) than NW in both age groups. In young adults, 21% (20/94) and 57% (45/79) of Spn6B + samples were detected by culture-enrichment only in NW and OPS respectively, indicating that culture-enrichment increased pneumococcal detection 2.7 times more in OPS samples than NW. In older adults, 35% (20/57), 64% (25/39) and 56% (5/9) of Spn6B + samples were detected by culture-enrichment only in NW, OPS and saliva respectively, indicating that culture-enrichment increased pneumococcal detection 1.1–1.8 times more in oropharyngeal (OPS and saliva) samples than in NW. Moreover, in case of the oropharyngeal niche, pneumococcal detection rates in OPS were 1.6 times higher than in saliva.

### Pneumococcal colonisation frequency and density with ageing in both nose and oropharynx

**Figure 1:**
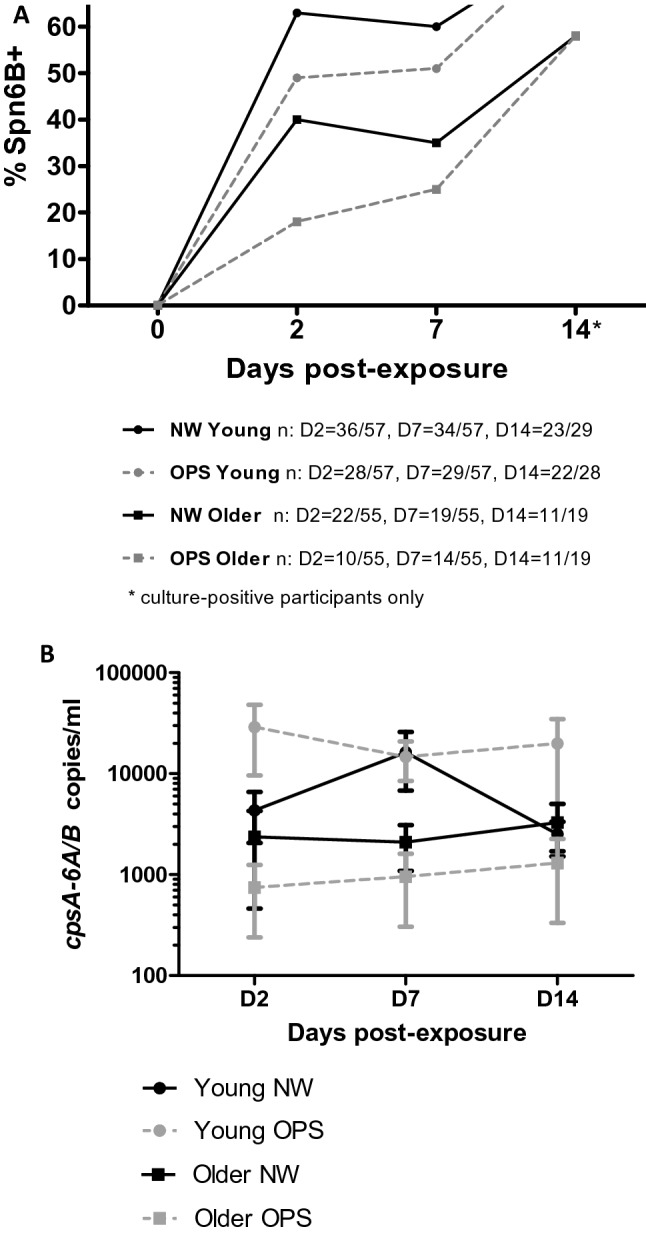
Experimental pneumococcal colonization in young (18–55 years, n = 57) and older adults (> 55 years, n = 55). (**A**) Frequency of Spn6B pneumococcus in nose (NW) and oropharynx (OPS) in both age groups. Detection of pneumococcal DNA was determined by multiplex qPCR. Participants with qPCR results positive for Spn6B (CT < 40) in the nose and oropharynx per day post-exposure per age group were: Young NW: D2 n = 36/57, D7 n = 34/57, D14 n = 23/29, Young OPS: D2 n = 28/57, D7 n = 29/57, D14 n = 22/28, Older NW: D2 n = 22/55, D7 n = 19/55, D14 n = 11/19, Older OPS: D2 n = 10/55, D7 n = 14/55, D14 n = 11/19. The number of volunteers with Spn6B + sample (CT < 40) in each day post-exposure is expressed as a percentage (%) of the total number of volunteers. Statistical significance was assessed by Fisher’s contingency test: Young vs Older adults: NW D2 *P* = 0.014, D7 *P* = 0.008, D14 *P* = 0.1931 and OPS D2 *P* = 0.0007, D7 *P* = 0.007, D14 *P* = 0.1946, Older adults NW vs OPS: D2 *P = 0.020. (**B**) Density of Spn6B pneumococcus in nose (NW) and oropharynx (OPS) in both age groups. Each time point represents the average density of SPN6B + per study day per niche. Data was log transformed. Graphs were created in GraphPad Prism version 5.0 for Windows, GraphPad Software, USA, www.graphpad.com.

To investigate the kinetics of experimental colonisation in both niches, we assessed the colonisation frequency and density of pneumococcal DNA in both age groups on days 2, 7 and 14 post pneumococcal exposure as shown in Fig. 1. For D14, only data from culture-positive participants in the older adults’ study was analysed to ensure comparability with the young vaccine study data. Pneumococcal colonisation frequency was significantly higher in young than older adults at all study days post exposure (Fig. 1A) in both NW (Young vs Older: Day 2 36/57 (63%) vs 22/55 (40%), *P* = 0.014, Day 7 34/57 (60%) vs 19/55 (34.5%), *P* = 0.008 and Day 14 23/29 (79.3%) vs 11/19 (57.9%), *P* = 0.1931) and OPS (Young vs Older: Day 2 28/57 (49.1%) vs 10/55 (18.2%), *P* = 0.0007, Day 7 29/57 (50.9%) vs 14/55 (25.5%), *P* = 0.007 and Day 14 22/28 (78.6%) vs 11/19 (57.9%), *P* = 0.1946). Comparing the two niches in both age groups separately, pneumococcal frequency was higher in the nose than the oropharynx at days 2 and 7 and similar at day 14 post pneumococcal exposure in both age groups (Fig. 1A, older adults NW 22/55 (40%) vs OPS 10/55 (18.2%) D2, *P* = 0.020).

Pneumococcal colonisation density was higher in young adults than in older adults for OPS (*P* = 0.008) but not for NW (*P* = 0.30) (Fig. 1B). Pneumococcal colonisation density was higher in NW than in OPS for older adults (*P* = 0.016) but not for young adults (*P* = 0.09) (Fig. 1B).

### Pneumococcal presence is higher in the nose (NW) than oropharynx (OPS) within and between young and older adults

**Figure 2:**
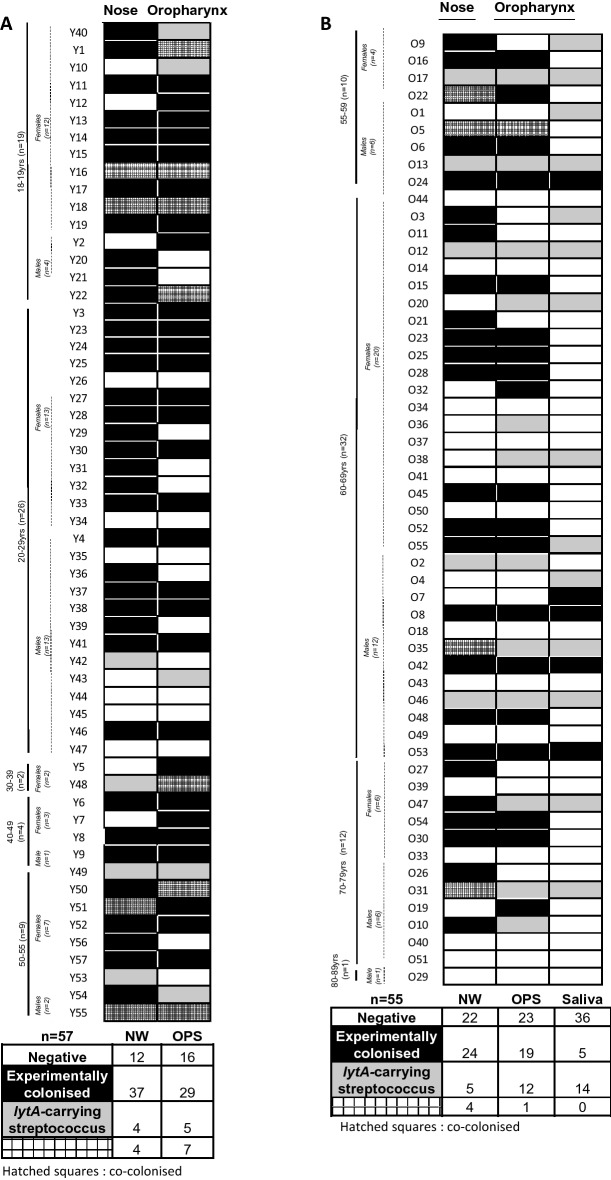
Heatmap showing individual nasal and oropharyngeal profiles in (**A**). young (18–55 years, n = 57) and (**B**) older (> 55 years, n = 55) adults. Participants are colour coded; white squares qPCR-negative, black squares Spn6B + , grey squares *lytA* + and hatched squares co-colonisers. Data is broken down by age group and gender. More Spn6B + participants were detected in nose (NW) than oropharynx (OPS) in both age groups (Young: NW n = 41 vs OPS n = 36, Older: NW n = 28 vs OPS n = 20). Pneumococcal presence in both NW (Young n = 41 vs Older n = 28, *P = 0.026) and OPS (Young n = 36 vs Older n = 20, **P = 0.008) was statistically significantly different between young and older adults (GLM model). In older adults, fewer Spn6B + participants were detected in saliva (n = 5). Heatmaps were created in Microsoft Excel (2016), Microsoft, USA, www.microsoft.com.

To compare overall pneumococcal detection rates between the nasal and oropharyngeal niches, an overall nasal (combined raw and culture-enriched NW) and oropharyngeal (combined raw and culture-enriched OPS) profile were created for each participant and plotted on a heat map as shown in Fig. 2. When extraction from raw and CE samples yielded different results, the positive result was retained regardless of the method. Participants with qPCR-negative samples on all study days were defined as negative (shown in white). Those with a Spn6B + sample on any study day were classified as experimentally colonised (shown in black). Participants with a *lytA* + but *cpsA-6A/B*—sample on any study day were classified as colonised with a *lytA*-carrying streptococcus (shown in grey). Participants with Spn6B + samples and *lytA* + (*cpsA-6A/B -)* samples on different study days were classified as co-colonised (shown with hatched shading).

The nose of 41/57 (72%) young adults was colonised with Spn6B (Fig. 2A). Using NW, 12/57 (21%) participants were negative, 37/57 (65%) experimentally colonised, 4/57 (7%) colonised with a *lytA*-carrying streptococcus and 4/57 (7%) co-colonised. The oropharynx of 36/57 (63%) young adults was colonised with Spn6B (Fig. 2A). Using OPS, 16/57 (28%) participants were negative, 29/57 (51%) experimentally colonised, 5/57 (9%) colonised with a *lytA*-carrying streptococcus and 7/57 (12%) co-colonised.

The carriage rate in older adults was lower than in younger adults. The nose of 28/55 (51%) older adults was colonised with Spn6B (Fig. 2B). Using NW, 22/55 (40%) participants were negative, 24/55 (44%) experimentally colonised, 5/55, (9%) colonised with a *lytA*-carrying streptococcus and 4/55 (7%) co-colonised. The oropharynx of 20/55 (36%) older adults, as assessed by OPS, was colonised with Spn6B (Fig. 2B). Using OPS, 22/55 (40%) participants were negative, 19/55 (35%) experimentally colonised, 12/55 (24%) colonised with a *lytA*-carrying streptococcus and 1/55 (2%) co-colonised. Because there were more participants colonised with *lytA*-carrying streptococci in their oropharynx in the older cohort, we analysed these samples by microarray. In all 12 cases, non-pneumococcal streptococci were identified (data not shown).

Using combined raw and culture-enrichment methods, higher pneumococcal presence was detected in the nose than the oropharynx in both age groups with statistical significance in older adults (Supplementary Table S4, *P* = 0.016). Pneumococcal presence was significantly different between young and older adults in both NW (Supplementary Table S4, *P* = 0.026) and OPS (Supplementary Table S4, *P* = 0.004).

### OPS is more sensitive than saliva for pneumococcal detection in older adults

Both OPS (described above, combined raw and culture-enriched OPS) and saliva (combined raw and culture-enriched saliva) samples were used to assess pneumococcal colonisation in the oropharynx of older adults. In saliva, 36/55 (65%) participants were negative, 5/55 (9%) experimentally colonised, 14/55 (25%) colonised with a *lytA*-carrying streptococcus and no co-colonised were detected. Therefore, overall, the oropharynx of 20/55 (36%) older adults, as assessed by both OPS and saliva, were colonised with Spn6B (Fig. 2B). Only 5 of these (25%) were detected in saliva compared to 19 (95%) in OPS, (Fig. 2B), indicating that in the older age group, saliva is a less sensitive method of assessing pneumococcal colonisation than OPS.

## Discussion

This study investigated whether the pneumococcal colonisation niche alters with increasing age following experimental human challenge. Our findings show that regardless of age, the nasal niche had the highest percentage of experimentally colonised participants.

Experimental colonisation involves the direct inoculation of pneumococcus into the nose and therefore may not precisely imitate natural colonisation dynamics. As little is known about natural pneumococcal transmission routes to the host, it is possible that the niche of first contact with transmitted pneumococci, in this case the nose, has a large effect on subsequent colonisation location. Nevertheless, we have described in a series of independent studies that participants who become colonised following inoculation develop a consistent colonisation episode of 1–3 weeks of similar density to natural colonisation^25,32^. Our findings agree with studies of natural colonisation where a higher incidence of pneumococcal growth is found in individuals’ nasopharyngeal samples compared with their oropharyngeal samples^16,33,34^.

Young adults showed a higher percentage of colonised participants than their older counterparts at each study day following inoculation, in both the nose and the oropharynx. The relationship between age and prevalence of colonisation has been well documented^35–37^, in agreement with our findings that prevalence of experimental colonisation decreases with age. This decrease did not reach statistical significance when samples were analysed using classical microbiology methods (*P* = 0.19), in keeping with the increased sensitivity of molecular methods.

Culture-enrichment of samples increased pneumococcal detection in both niches in both age groups. This extra step has been shown previously to increase pneumococcal detection in saliva^21,38^ and our group now uses it routinely when analysing clinical trial samples. Although labour-intensive, we believe that its added value justifies recommending it as standard practice in combination with analysing raw samples^39^.

The strength of our study is the collection of paired longitudinal nasal and oropharyngeal samples before and after pneumococcal inoculation of a known strain. Coupled with culture enrichment and molecular methods for capsular polysaccharide-specific detection, this allows the precise determination of the frequency and density of bacteria for each study day and niche, according to age. A weakness of our work is the lack of saliva in the young cohort and that collection of samples in the older and young cohorts was conducted during different studies. However, the strain used for inoculation as well as the methods for inoculation, nasal wash collection and processing remained identical, allowing for direct comparison of cohorts as done previously^24^. A further limitation could extend to the methods used for nasal sampling and saliva collection. Nasal wash is more comfortable and more sensitive than nasopharyngeal swab for pneumococcal detection in adults^10^, however it is not always feasible outside of a clinic setting. It may be that we would not have seen such a difference between the nose and oropharynx if we had used nasopharyngeal swabs. Our saliva detection levels were also much lower than those reported elsewhere when a spitting method was used for sample collection^21,39^. In older adults, with drier mouths, low sample volumes could be obscured using the Salivette device.

Our results indicate that age-related host factors could affect experimental colonisation prevalence in these age groups. The percentage of participants showing experimental carriage fell between days 2 and day 14 in the nose of older age group, as assessed by NW. This could be evidence of experimental pneumococcal colonisation clearance, which is influenced by host immune responses such as local phagocytic function and acute mucosal inflammatory responses^39^ as well as mechanisms known to be affected by immunosenescence (toll like receptors and reduction in the function of host signalling pathways)^40^. Unlike pneumococcal colonisation prevalence, pneumococcal density in NW was unaffected by sampling age after experimental inoculation Several natural pneumococcal colonisation studies have found an opposite relationship between nasopharyngeal colonisation density and age indicating higher pneumococcal densities in younger subjects^41–43^.

It has been established that the complexity and diversity of the microbiome increases from the nasopharynx to the oropharynx to saliva^44^. Investigation of NW, OPS and saliva samples in this study showed a higher percentage of participants colonised with a *lytA*-carrying streptococcus in OPS (and saliva in older adults) in contrast to NW in both age groups. Due to higher levels of species diversity within the oral cavity, and the capacity of pneumococci to exchange genes with other streptococci, the use of *lytA* and *cps* genes as PCR targets needs to be treated with caution. Positive *lytA* PCR results may indicate the presence of non-pneumococcal species in addition to pneumococcal species, leading to false positives^45^, which was indeed the case here. Recent evidence has also emerged of the presence of non-pneumococcal streptococci containing capsular genes^46,47^. In addition, in our experience the addition of saliva sampling in older adults was not beneficial as it is less sensitive when compared with OPS.

In summary, this study has shown that the optimal sampling niche to detect experimental pneumococcal colonisation is the nose regardless of age, as assessed by NW. However, individuals show different colonised niches, so reducing sampling to only the nose would exclude detection of pneumococcal colonisation in some patients (both young and older adults). Future studies could investigate the site of pneumococcal colonisation over a longer time-period following experimental inoculation. The current study examined a short period of time, and studies in the literature of natural carriage are snapshots in time.

## Supplementary Information

Supplementary Information.

## Data Availability

The datasets generated during and/or analysed during the current study are available from the corresponding author on reasonable request.

## References

[1] Costello EK, Stagaman K, Dethlefsen L, Bohannan BJM, Relman DA (2012). The application of ecological theory toward an understanding of the human microbiome. Science.

[2] Hales C (2020). Symptoms associated with influenza vaccination and experimental human pneumococcal colonisation of the nasopharynx. Vaccine.

[3] Trimble A (2020). Pneumococcal colonisation is an asymptomatic event in healthy adults using an experimental human colonisation model. PLoS ONE.

[4] Bogaert D, De Groot R, Hermans PW (2004). Streptococcus pneumoniae colonisation: The key to pneumococcal disease. Lancet. Infect. Dis..

[5] Simell B (2012). The fundamental link between pneumococcal carriage and disease. Expert Rev. Vaccines.

[6] Melegaro A, Gay NJ, Medley GF (2004). Estimating the transmission parameters of pneumococcal carriage in households. Epidemiol. Infect..

[7] O’Brien KL, Nohynek H (2003). & The Who Pneumococcal Vaccine Trials Carriage Working, G. Report from a WHO Working Group: standard method for detecting upper respiratory carriage of Streptococcus pneumoniae. Pediatr. Infect. Dis. J..

[8] Satzke C (2013). Standard method for detecting upper respiratory carriage of Streptococcus pneumoniae: Updated recommendations from the World Health Organization Pneumococcal Carriage Working Group. Vaccine.

[9] Arguedas A (2020). Upper respiratory tract colonization with *Streptococcus pneumoniae* in adults. Expert Rev. Vacc..

[10] Gritzfeld JF, Roberts P, Roche L, El Batrawy S, Gordon SB (2011). Comparison between nasopharyngeal swab and nasal wash, using culture and PCR, in the detection of potential respiratory pathogens. BMC. Res. Notes.

[11] Bomar L, Brugger SD, Lemon KP (2018). Bacterial microbiota of the nasal passages across the span of human life. Curr. Opin. Microbiol..

[12] Murad C (2019). Pneumococcal carriage, density, and co-colonization dynamics: A longitudinal study in Indonesian infants. Int. J. Infect. Dis..

[13] Goldblatt D (2005). Antibody responses to nasopharyngeal carriage of Streptococcus pneumoniae in adults: A longitudinal household study. J. Infect. Dis..

[14] Heinsbroek E (2015). Persisting high prevalence of pneumococcal carriage among HIV-infected adults receiving antiretroviral therapy in Malawi: A cohort study. AIDS.

[15] Numminen E (2015). Climate induces seasonality in pneumococcal transmission. Sci. Rep..

[16] Watt JP (2004). Nasopharyngeal versus oropharyngeal sampling for detection of pneumococcal carriage in adults. J. Clin. Microbiol..

[17] Becker-Dreps S (2015). Pneumococcal carriage and vaccine coverage in retirement community residents. J. Am. Geriatr. Soc..

[18] Flamaing J, Peetermans WE, Vandeven J, Verhaegen J (2010). Pneumococcal colonization in older persons in a nonoutbreak setting. J. Am. Geriatr. Soc..

[19] Zanella RC (2019). Nasopharyngeal carriage of *Streptococcus pneumoniae*, *Haemophilus influenzae*, and *Staphylococcus aureus* in a Brazilian elderly cohort. PLoS ONE.

[20] Smith EL (2020). Upper airways colonisation of Streptococcus pneumoniae in adults aged 60 years and older: A systematic review of prevalence and individual participant data meta-analysis of risk factors. J. Infect..

[21] Krone CL (2015). Carriage of *Streptococcus pneumoniae* in aged adults with influenza-like-illness. PLoS ONE.

[22] van Deursen AM, van den Bergh MR, Sanders EA (2016). Carriage of *Streptococcus* pneumoniae in asymptomatic, community-dwelling elderly in the Netherlands. Vaccine.

[23] Gritzfeld JF (2013). Experimental human pneumococcal carriage. J. Vis. Exp. JoVE.

[24] Adler H (2020). Experimental human pneumococcal colonisation in older adults is feasible and safe, not immunogenic. Am. J. Respir. Crit. Care Med..

[25] Ferreira DM (2013). Controlled human infection and rechallenge with *Streptococcus pneumoniae* reveals the protective efficacy of carriage in healthy adults. Am. J. Respir. Crit. Care Med..

[26] Collins AM (2015). First human challenge testing of a pneumococcal vaccine double-blind randomized controlled trial. Am. J. Respir. Crit. Care Med..

[27] Adler H (2019). Pneumococcal colonization in healthy adult research participants in the conjugate vaccine era, United Kingdom, 2010–2017. J. Infect. Dis..

[28] Marrie TJ, Tyrrell GJ, Majumdar SR, Eurich DT (2018). Effect of age on the manifestations and outcomes of invasive pneumococcal disease in adults. Am. J. Med..

[29] Carvalho Mda G (2007). Evaluation and improvement of real-time PCR assays targeting lytA, ply, and psaA genes for detection of pneumococcal DNA. J. Clin. Microbiol..

[30] Azzari C (2010). Realtime PCR is more sensitive than multiplex PCR for diagnosis and serotyping in children with culture negative pneumococcal invasive disease. PLoS ONE.

[31] German EL (2019). Protective effect of PCV vaccine against experimental pneumococcal challenge in adults is primarily mediated by controlling colonisation density. Vaccine.

[32] Gritzfeld JF (2014). Density and duration of experimental human pneumococcal carriage. Clin. Microbiol. Infect..

[33] Lieberman D (2006). Nasopharyngeal versus oropharyngeal sampling for isolation of potential respiratory pathogens in adults. J. Clin. Microbiol..

[34] Farrar JL (2020). Limited added value of oropharyngeal swabs for detecting pneumococcal carriage in adults. Open Forum Infect. Dis..

[35] Grant LR (2016). Impact of the 13-valent pneumococcal conjugate vaccine on pneumococcal carriage among American Indians. Pediatr. Infect. Dis. J..

[36] Mackenzie GA, Leach AJ, Carapetis JR, Fisher J, Morris PS (2010). Epidemiology of nasopharyngeal carriage of respiratory bacterial pathogens in children and adults: Cross-sectional surveys in a population with high rates of pneumococcal disease. BMC Infect. Dis..

[37] Scott JR (2012). Impact of more than a decade of pneumococcal conjugate vaccine use on carriage and invasive potential in Native American communities. J. Infect. Dis..

[38] Wyllie AL (2016). Molecular surveillance on *Streptococcus pneumoniae* carriage in non-elderly adults; little evidence for pneumococcal circulation independent from the reservoir in children. Sci. Rep..

[39] Nikolaou E (2021). Experimental human challenge defines distinct pneumococcal kinetic profiles and mucosal responses between colonized and non-colonized adults. MBio.

[40] Krone CL, van de Groep K, Trzciński K, Sanders EA, Bogaert D (2014). Immunosenescence and pneumococcal disease: An imbalance in host-pathogen interactions. Lancet Respir. Med..

[41] Roca A (2012). Effect of age and vaccination with a pneumococcal conjugate vaccine on the density of pneumococcal nasopharyngeal carriage. Clin. Infect. Dis..

[42] Trzciński K (2013). Superiority of trans-oral over trans-nasal sampling in detecting *Streptococcus pneumoniae* colonization in adults. PLoS ONE.

[43] Sutcliffe CG (2019). Association of laboratory methods, colonization density, and age with detection of *Streptococcus pneumoniae* in the nasopharynx. Am. J. Epidemiol..

[44] Tavares DA (2019). Identification of *Streptococcus pneumoniae* by a real-time PCR assay targeting SP2020. Sci Rep.

[45] Johnston C (2010). Detection of large numbers of pneumococcal virulence genes in streptococci of the mitis group. J. Clin. Microbiol..

[46] Carvalho Mda G (2013). Non-pneumococcal mitis-group streptococci confound detection of pneumococcal capsular serotype-specific loci in upper respiratory tract. PeerJ.

[47] Carvalho MG (2012). Potential nonpneumococcal confounding of PCR-based determination of serotype in carriage. J. Clin. Microbiol..

