# Supplementary material for: The nose is the best niche for detection of experimental pneumococcal colonisation in adults of all ages, using nasal wash
Source: Sci Rep. 2021 Sep 14;11:18279. doi: 10.1038/s41598-021-97807-1 (PMC8440778; doi:10.1038/s41598-021-97807-1)
Supplement: Supplementary file 1 — Supplementary Information. [file 41598_2021_97807_MOESM1_ESM.docx]

**Supplementary Tables**

**Table S1. Participants’ demographic data.** One hundred and twelve participants: 57 aged 18-55 years and 55 aged >55 years.

|  | **Young adults (n=57)** | **Older adults (n=55)** |
| --- | --- | --- |
| **Mean age in years (SD)** | 27.6 (12.1) | 65.5 (5.8) |
| **No. Female/Male** | 36/21 | 30/25 |

**Table S2. Distribution of SPN6B+ in A.** NW and **B.** OPS samples per day post pneumococcal exposure in young adults.

| 1. NW Spn6B+ (n=94) | | | | |
| --- | --- | --- | --- | --- |
| Study day | **Total / Day** | **RAW only** | **CE only** | **Both** |
| D2 (n=57) | 36/57 (63%) | 6/36 (17%) | 7/36 (20%) | 23/36 (64%) |
| D7 (n=57) | 34/57 (60%) | 4/34 (12%) | 8/34 (24%) | 22/34 (65%) |
| D14 (n=33) | 24/33 (73%) | 3/24 (13%) | 5/24 (21%) | 16/24 (67%) |
| Total | 94/147 (64%) | 13/94 (14%) | 20/94 (21%) | 61/94 (65%) |

| B.  OPS Spn6B+ (n=79) | | | | |
| --- | --- | --- | --- | --- |
| Study Day | **Total / Day** | **RAW only** | **CE only** | **Both** |
| D2 (n=57) | 28/57 (49%) | 0/28 (0%) | 19/28 (68%) | 9/28 (32%) |
| D7 (n=57) | 29/57 (51%) | 0/29 (0%) | 14/29 (48%) | 15/29 (52%) |
| D14 (n=32) | 22/32 (69%) | 0/22 (0%) | 12/22 (55%) | 10/22 (45%) |
| Total | 79/146 (54%) | 0/79 (0%) | 45/79 (57%) | 34/79 (43%) |

**Table S3. Distribution of SPN6B+ in A.** NW, **B.** OPS and **C.** Saliva samples per day post pneumococcal exposure in older adults.

| 1. NW Spn6B+ (n=57) | | | | |
| --- | --- | --- | --- | --- |
| Study Day | **Total/Day** | **RAW only** | **CE only** | **Both** |
| D2 (n=55) | 22/55 (40%) | 3/22 (14%) | 8/22 (36%) | 11/22 (50%) |
| D7 (n=55) | 19/55 (35%) | 0/19 (0%) | 7/19 (37%) | 12/19 (63%) |
| D14 (n=53) | 16/53 (30%) | 0/16 (0%) | 5/16 (31%) | 11/16 (69%) |
| Total | 57/163 (35%) | 3/57 (5%) | 20/57 (35%) | 34/57 (60%) |

| 1. OPS Spn6B+ (n=39) | | | | |
| --- | --- | --- | --- | --- |
| Study Day | **Total/Day** | **RAW only** | **CE only** | **Both** |
| D2 (n=55) | 10/55 (18%) | 1/10 (10%) | 8/10 (80%) | 1/10 (10%) |
| D7 (n=55) | 14/55 (25%) | 0/14 (0%) | 8/14 (57%) | 6/14 (43%) |
| D14 (n=53) | 15/53 (28%) | 0/15 (0%) | 9/15 (60%) | 6/15 (40%) |
| Total | 39/163 (24%) | 1/39 (3%) | 25/39 (64%) | 13/39 (33%) |

| 1. SAL Spn6B+ (n=9) | | | | |
| --- | --- | --- | --- | --- |
| Study Day | **Total/Day** | **RAW only** | **CE only** | **Both** |
| D2 (n=55) | 4/55 (7%) | 0/4 (0%) | 3/4 (75%) | 1/4 (25%) |
| D7 (n=54) | 3/54 (6%) | 0/3 (0%) | 1/3 (33%) | 2/3 (67%) |
| D14 (n=52) | 2/52 (4%) | 0/2 (0%) | 1/2 (50%) | 1/2 (50%) |
| Total | 9/161 (6%) | 0/9 (0%) | 5/9 (56%) | 4/9 (44%) |

**Table S4. Association of SPN6B+ in A.** young and **B.** older adults between the two niches and in the **C.** nose (NW) and **D.** oropharynx (OPS) between the two age groups. The interaction between niche and age was statistically significant (P=0.015).

| 1. Young adults (n=57) |  |  |  |
| --- | --- | --- | --- |
| **NW Spn6B+** | **OPS Spn6B+** | **Rate difference** | **P value** |
| 41(72%) | 36(63%) | 8.89(-4.12,21.91) | 0.180 |
| 1. Older Adults (n=55) |  |  |  |
| **NW Spn6B+** | **OPS Spn6B+** | **Rate difference** | **P value** |
| 28(51%) | 20(36%) | 14.48(2.76,26.20) | 0.016 |
| 1. NW Spn6B+ |  |  |  |
| **Young (n=57)** | **Older (n=55)** | **Rate difference** | **P value** |
| 41(72%) | 28(51%) | 20.03(2.36,37.71) | 0.026 |
| 1. OPS Spn6B+ |  |  |  |
| **Young (n=57)** | **Older (n=55)** | **Rate difference** | **P value** |
| 36(63%) | 20(36%) | 26.36(8.43,44.30) | 0.004 |
